# Flavonoid-mediated immunomodulation of human macrophages involves key metabolites and metabolic pathways

**DOI:** 10.1038/s41598-019-51113-z

**Published:** 2019-10-17

**Authors:** Luís F. Mendes, Vítor M. Gaspar, Tiago A. Conde, João F. Mano, Iola F. Duarte

**Affiliations:** CICECO – Aveiro Institute of Materials, Department of Chemistry, University of Aveiro, 3810-193 Aveiro, Portugal

**Keywords:** Biochemistry, Systems biology

## Abstract

The ability of flavonoids to attenuate macrophage pro-inflammatory activity and to promote macrophage-mediated resolution of inflammation is still poorly understood at the biochemical level. In this study, we have employed NMR metabolomics to assess how therapeutically promising flavonoids (quercetin, naringenin and naringin) affect the metabolism of human macrophages, with a view to better understand their biological targets and activity. *In vitro*-cultured human macrophages were polarized to the pro-inflammatory M1 phenotype, through incubation with LPS + IFN-γ, and subsequently treated with each flavonoid. The metabolic signatures of pro-inflammatory polarization and of flavonoid incubations were then characterized and compared. The results showed that all flavonoids modulated the cells endometabolome with the strongest impact being observed for quercetin. Many of the flavonoid-induced metabolic variations were in the opposite sense to those elicited by pro-inflammatory stimulation. In particular, the metabolic processes proposed to reflect flavonoid-mediated immunomodulation of macrophages included the downregulation of glycolytic activity, observed for all flavonoids, anti-inflammatory reprogramming of the TCA cycle (mainly quercetin), increased antioxidant protection (quercetin), osmoregulation (naringin), and membrane modification (naringenin). This work revealed key metabolites and metabolic pathways involved in macrophage responses to quercetin, naringenin and naringin, providing novel insights into their immunomodulatory activity.

## Introduction

Chronic inflammation has been implicated in the pathogenesis of multiple diseases, such as arthritis, cancer, colitis and atherosclerosis^1^, as well as in the rejection of transplanted organs^2^ or medical implants^3^. Therefore, finding novel approaches to promote appropriate resolution of inflammatory processes is of utmost importance. Macrophages play a key role in all stages of inflammation, mainly through phagocytosis and the production of chemokines, cytokines and growth factors^4^. Bearing an immense phenotypic and functional plasticity, these cells exhibit a continuum of polarization states, triggered by different microenvironment cues, whose regulation critically defines beneficial or detrimental outcomes in inflammatory responses^5^. Under specific *in vitro* conditions, two main phenotypes have been identified: pro-inflammatory M1 macrophages, arising from stimulation with bacterial lipopolysaccharide (LPS)/interferon-γ (IFN-γ), and anti-inflammatory or reparative M2 macrophages, which comprise several subtypes and result from stimulation with interleukins like IL-4/IL-13^6^. Although this framework is a simplified view of macrophage plasticity *in vivo*^7^, it is widely accepted that the balance between M1-like pro-inflammatory and M2-like anti-inflammatory macrophages is crucial for inflammation resolution and tissue homeostasis^1^. Hence, the modulation of macrophage polarization is currently viewed as a promising strategy for treating inflammatory diseases, as well as in the context of tissue engineering and regenerative medicine^8,9^.

Several studies have shown that natural compounds, including flavonoids, have the ability to instruct macrophages from pro- to anti-inflammatory phenotypes, potentially contributing to the resolution of pre-established inflammatory processes^10,11^. The anti-inflammatory activity of flavonoids has been attributed to multiple mechanisms, including inactivation of the nuclear factor κ-light-chain-enhancer of activated B cells (NF-κB), modulation of the mitogen-activated protein kinase (MAPK) and arachidonic acid pathways, inhibition of phosphatidylinositide 3-kinases/protein kinase B (PI3K/AkT) and mammalian target of rapamycin complex 1 (mTORC1) signaling, downregulation of pro-inflammatory gene expression and cytokine synthesis, and inhibition of reactive oxygen species (ROS) and nitric oxide (NO) production^12^. However, the biological mechanisms underlying flavonoid-mediated modulation of macrophage phenotypes in inflammation are poorly understood and their real therapeutic potential underappreciated.

Metabolism has recently been uncovered as a vital axis of macrophage phenotypic and functional regulation^13,14^. Activation-dependent alterations have been linked to numerous metabolic pathways, including glycolysis, amino acid metabolism, tricarboxylic acids (TCA) cycle and oxidative phosphorylation (OxPhos), and macrophage metabolism has emerged as a potentially valuable therapeutic target to control inflammation^15–19^. Hence, studying the effect of flavonoids on macrophage metabolism is crucial to better elucidate their mechanisms of action and potentiate their possible therapeutic use as immunomodulators. Metabolomics holds great potential to investigate metabolites and metabolic pathways involved in macrophage immunomodulation, as shown by studies involving macrophage exposure to various stimuli, such as bacterial LPS^20^, atmospheric pollutants^21^, nanoparticles^22,23^ or natural compounds^24,25^. However, to our knowledge, a comprehensive characterization of the metabolomic responses following flavonoid administration to human macrophages has not been previously reported.

In this study, NMR-based metabolomics was employed to assess macrophage metabolic reprogramming upon exposure to quercetin, naringenin, and naringin, which are abundant flavonoids in fruits and vegetables with well-known anti-inflammatory activity but poorly investigated metabolic modulation in macrophages. This research is expected to advance current understanding of these flavonoids mode of action, potentially supporting their use as dietary supplements and/or immunomodulatory drugs.

## Methods

### Test compounds and reagents

Stock solutions of quercetin (97% purity, Alfa Aesar, Ward Hill, MA, USA), naringenin (≥95% purity, Merck - Sigma Aldrich, St. Louis, MO, USA) and naringin (≥95% purity, Merck - Sigma Aldrich, St. Louis, MO, USA), the structures of which are displayed in Supporting Information Fig. S1, were prepared with 99.5% DMSO at a concentration of 80 mM. Phorbol 12-myristate 13-acetate (PMA, Merck - Sigma Aldrich, St. Louis, MO, USA) was prepared in 99.5% of DMSO at a concentration of 100 μg/mL. Powdered LPS (Merck - Sigma Aldrich, St. Louis, MO, USA) was dissolved in Gibco Water for Injection (WFI) at a concentration of 1 mg/mL. IFN-γ (BioLegend, San Diego, CA, USA) was kept in the original formulation at a concentration of 0.1 mg/mL. All solutions were kept at −20 °C and protected from light.

### Macrophage differentiation and polarization

Human monocytic THP-1 cells obtained from the American Type Culture Collection (ATCC TIB-202, Manassas, VA, USA) were cultured in suspension at 37 °C and a 5% CO_2_ atmosphere, in Roswell Park Memorial Institute Medium (RPMI 1640, Gibco - Thermo Fischer Scientific, Waltham, MA, USA) supplemented with 2.5 g/L of sodium bicarbonate (Merck - Sigma Aldrich, St. Louis, MO, USA) and containing 10% of heat inactivated fetal bovine serum (FBS) and 1% of an antibiotic/antimycotic solution (penicillin and streptomycin). The cells were maintained in suspension in non-adherent 100 mm-diameter petri-dishes. When a high cellular density was reached, the cells were sub-cultured and differentiated into macrophages via a 24 h incubation with 50 ng/mL PMA, followed by a second 24 h incubation with fresh medium, after which >90% monocytes were fully differentiated into adherent macrophages (M0).

Generation of pro-inflammatory M1 macrophages was performed through incubation with 100 ng/mL of LPS and 20 ng/mL of IFN-γ for 24 h, as previously reported^6,26^. To assess macrophage polarization, cytokine quantification was performed in the cells medium supernatants, using the LEGENDplex Human M1/M2 Macrophage Panel (10-plex, BioLegend, San Diego, CA, USA) comprising TNF-α IL-1β, IL-1RA, IL-6, IL-10, IL-12p40, IL-12p70, IL-23, CCL17 and CXCL10. Samples were prepared as recommended by the manufacturer and analyzed in a flow cytometer (BD Accuri C6 plus, BD Biosciences, Franklin Lakes, NJ, USA), configured to acquire 4 × 10^3^ events in the region of interest (ROI) corresponding to the different beads and respective analytes. Calibration curves were recorded for each analyte and data were post-processed in the proprietary LEGENDplex software. As expected, macrophages stimulated with LPS and IFN-γ increased the production of several pro-inflammatory cytokines (Supporting Information Fig. S2), confirming M1 polarization.

### Macrophage viability upon flavonoid treatment

Flavonoid cytotoxicity was assessed through the Alamar Blue assay. Specifically, medium from THP-1 derived macrophages, cultured in 96-well plates, was replaced with fresh medium containing different concentrations of the tested compounds (0, 20, 40, 60, 80, 100, 150 and 200 μM) and cells were incubated for additional 24 h, before incubation with Alamar Blue according to the manufacturer’s instructions. The samples were then analyzed in a fluorescence microplate reader (Synergy HTX, BioTek instruments, Winooski, VT, USA) (λEx = 540 nm; λEm = 600 nm). Based on the cell viability results (Supporting Information Fig. S3), the highest non-cytotoxic concentrations of each compounds were selected for subsequent assays: quercetin 60 μM, naringenin 100 μM, naringin 200 μM.

### Cellular incubations and sample collection for metabolomics analysis

**Figure 1:**
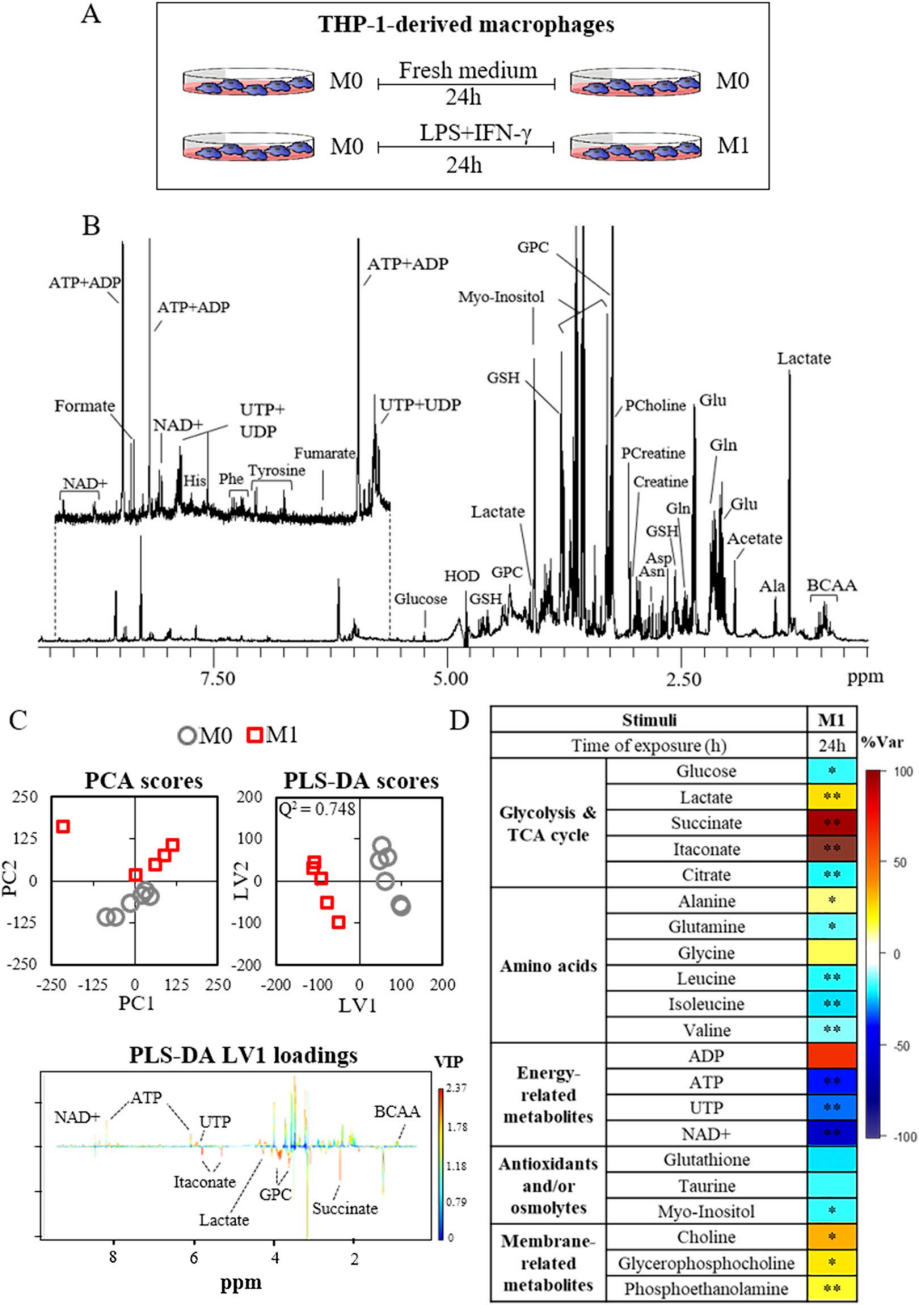
(**A**) Schematic representation of unstimulated (M0 controls) and pro-inflammatory (M1) THP-1-derived macrophages; (**B**) Typical ^1^H NMR spectrum obtained for polar extracts of THP-1-derived macrophages; (**C**) Multivariate analysis of spectral data from M0 and M1 macrophages; D) Heatmap of metabolic effects induced by LPS + IFN-γ, where the color scale represents the percentage of variation in M1 *vs*. M0 macrophages (*p < 0.05, **p < 0.01). The scores plots were prepared using Microsoft Excel 2013. The loadings profile and the heatmap were generated using R software version 3.4.1, R Core Team (2017). R: A language and environment for statistical computing. R Foundation for Statistical Computing, Vienna, Austria. http://www.R-project.org/.

**Figure 2:**
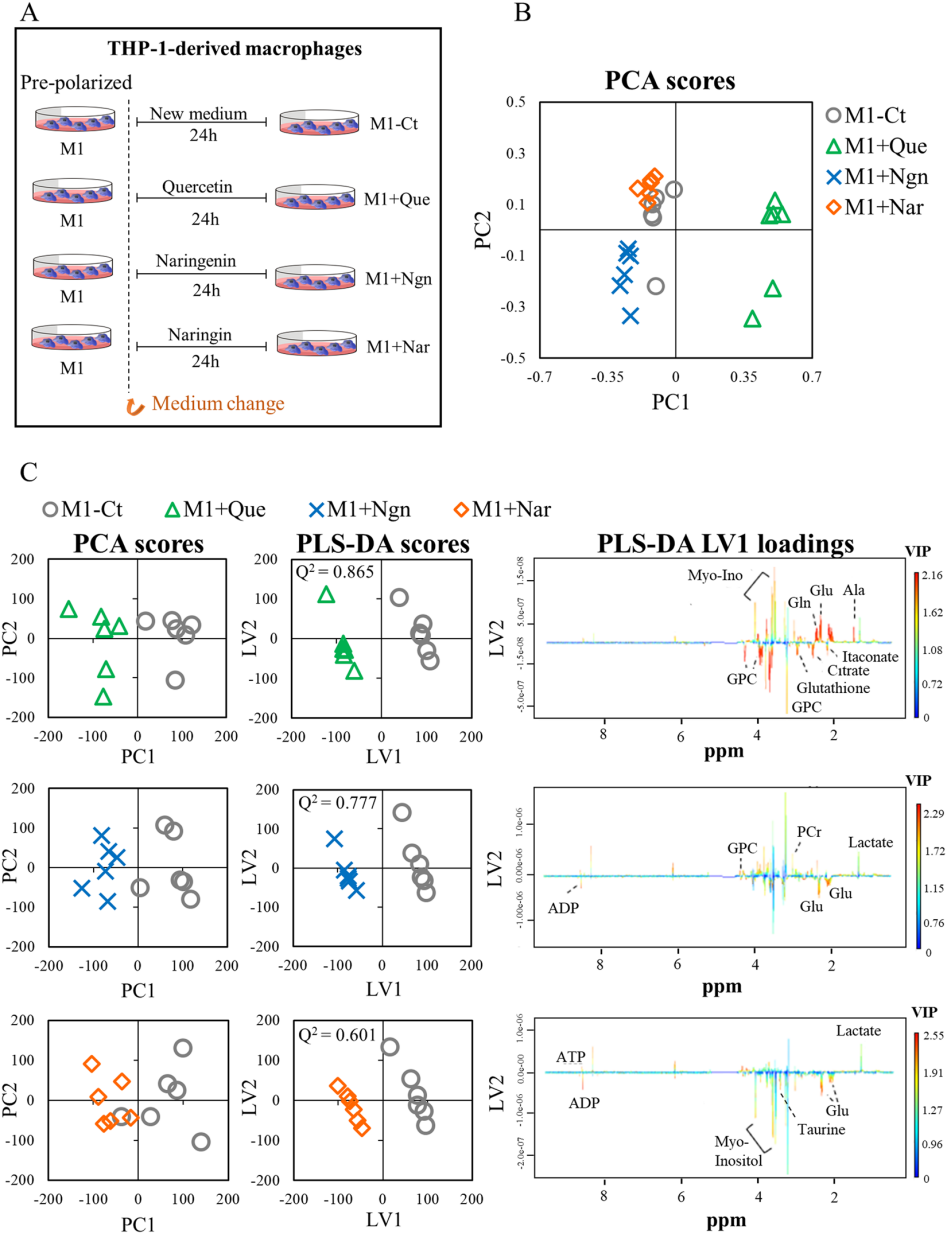
(**A**) Schematic representation of flavonoid treatment of pre-polarized M1 macrophages; (**B**) Scores scatter plot obtained by PCA of spectral data from flavonoid-treated and control (M1-Ct) groups; (**C**) Multivariate analysis performed for pairwise comparisons between macrophages incubated with each flavonoid and the control group. The scores plots were prepared using Microsoft Excel 2013. The loadings profiles were generated using R software version 3.4.1, R Core Team (2017). R: A language and environment for statistical computing. R Foundation for Statistical Computing, Vienna, Austria. http://www.R-project.org/.

Macrophages seeded in 100 mm diameter petri-dishes at a density of 1 × 10^6^ cells/mL were stimulated with LPS and IFN-γ as described above and schematically represented in Fig. 1A. After 24 h, the culture medium was discarded and the cells were incubated with fresh complete medium containing each flavonoid at selected concentrations, or with fresh complete medium alone (controls M1-Ct), for additional 24 h, as represented in Fig. 2A. Three independent assays with 2 replicates each were performed, giving a total of 6 samples per condition. For preparing the samples, the medium was removed and stored at −80 °C, the cells were washed 4 times with 10 mL of cold PBS, and extracted using a biphasic extraction protocol with methanol:chloroform:water (1:1:0.7), as previously described^27^. The resulting polar extracts were then dried under vacuum in a speedvac concentrator and stored at −80 °C.

### NMR data acquisition and processing

At the time of NMR analysis, the dried aqueous extracts were suspended in 600 μL of deuterated phosphate buffer (PBS 100 mM, pH 7) containing 0.1 mM of 3-(trimethylsilyl)propionic acid (TSP-*d*_4_), and 550 μL of each sample were transferred to 5 mm NMR tubes. All samples were analyzed in a Bruker Avance III HD 500 NMR spectrometer (University of Aveiro, Portuguese NMR Network) operating at 500.13 MHz for ^1^H observation, at 298 K. Standard 1D ^1^H spectra with water presaturation (pulse program ‘noesypr1d’, Bruker library) were recorded with 32 k points, 7002.801 Hz spectral width, a 2 s relaxation delay and 2048 scans. Spectral processing in TopSpin 4.0.3 (Bruker BioSpin, Rheinstetten, Germany) comprised cosine multiplication (ssb 2), zero-filling to 64 k data points, manual phasing and baseline correction, and calibration to the TSP-*d*_4_ signal (δ 0 ppm). Two-dimensional NMR spectra, namely ^1^H-^1^H TOCSY, *J*-resolved and ^1^H-^13^C HSQC spectra, were also recorded for selected samples to aid metabolite identification. Metabolite assignment was based on matching 1D and 2D spectral information to reference spectra available in Chenomx (Edmonton, Canada), BBIOREFCODE-2–0–0 (Bruker Biospin, Rheinstetten, Germany) and HMDB^28^.

### Multivariate analysis and integration of NMR spectra

Following spectra normalization by total area (excluding the suppressed water region and residual solvent signals), data matrices were uploaded into SIMCA-P 11.5 (Umetrics, Umeå, Sweden) and scaled to unit variance (UV) to give all variables equal variance. Principal Component Analysis (PCA) and Partial Least Squares- Discriminant Analysis (PLS-DA) were then applied and the results were visualized through factorial coordinates (‘scores’) and factorial contributions (‘loadings’) colored according to variable importance to the projection (VIP). For PLS-DA models, Q^2^ and R^2^ values, respectively reflecting predictive capability and explained variance, obtained from sevenfold internal cross validation, were used to assess the robustness of group discrimination. To provide a quantitative measurement of metabolic variations, spectral integration and total area normalization of selected signals were carried out in Amix-Viewer 3.9.15 (Bruker Biospin, Rheinstetten, Germany). For each metabolite, the percentage of variation in stimulated samples was calculated relative to respective controls, along with the effect size (ES)^29^ and statistical significance (p-value). The variations with larger magnitude (|ES| >0.5) were expressed in a heatmap. Loadings profiles and heatmap figures were generated using the R software version 3.4.1. (R Core Team (2017). R: A language and environment for statistical computing. R Foundation for Statistical Computing, Vienna, Austria. http://www.R-project.org/).

### Univariate statistics

The results were shown as mean ± SD, with n ≥3. When comparisons were made between several groups, the statistical significance was assessed via one-way ANOVA, with a Sidak multiple comparison test. Otherwise, the Student’s t test for small sample size was used. Statistical differences were indicated as *p < 0.05 and **p < 0.01.

## Results and Discussion

### Macrophage stimulation with LPS/IFN-γ induces pro-inflammatory metabolic reprogramming

Metabolic profiling of THP-1-derived macrophages by ^1^H NMR spectroscopy allowed the identification of 45 intracellular metabolites, comprising sugars, organic acids, amino acids, nucleotides, osmolytes, antioxidant metabolites and membrane-related compounds (Fig. 1B and Supporting Information Fig. S4 and Table S1). As a first approach to identify the metabolic effects of pro-inflammatory stimulation, multivariate analysis (MVA) was applied to the spectral data collected for uncommitted cells (M0 macrophages) and cells incubated with LPS + IFN-γ (M1 macrophages). As shown in Fig. 1C, these two groups were clearly separated in the PCA scores scatter plot and discriminated by PLS-DA with high robustness (Q^2^ 0.748). The corresponding LV1 loadings revealed the main variations responsible for such discrimination, which were then verified through spectral integration of individual metabolite signals. A heatmap representation of all relevant intracellular variations in M1 relative to M0 macrophages is displayed in Fig. 1D (detailed data presented in Supporting Table S2). M1 macrophages exhibited decreased glucose and increased lactate levels, suggesting enhanced glycolytic activity, in accordance with previous studies on murine^15,30,31^ and human^20,32,33^ macrophages stimulated with LPS or LPS + IFN-γ. Indeed, upregulation of glycolysis is considered a major hallmark of pro-inflammatory macrophage activation, as it allows for rapid energy generation and has been linked to the production of pro-inflammatory cytokines^14^. Reprogramming of macrophage TCA cycle is another well-established metabolic signature of pro-inflammatory stimulation, which comprises induction of the Immune Responsive Gene 1 (Irg1) with consequent inhibition of isocitrate dehydrogenase (IDH) and production of itaconate from citrate-derived cis-aconitate, as well as altered succinate processing through succinate dehydrogenase (SDH)^34,35^. In brief, succinate is considered a pro-inflammatory metabolite, mainly through stabilization of HIF-1α, which promotes glycolysis and transcription of IL-1β^15^, while the production of itaconate has been highlighted as a counteracting anti-inflammatory reaction to avoid persistence of inflammation^36^. Accordingly, we observed significantly increased levels of succinate and itaconate, together with decreased levels of citrate, in M1 macrophages relative to controls. Moreover, glutamine was decreased, therefore supporting its possible use for itaconate production, as demonstrated in LPS-stimulated murine macrophages^18^. As for other amino acids, we found decreased levels of leucine, isoleucine and valine, which agrees with recent data where the catabolism of branched chain amino acids was postulated to be involved in M1 macrophage activation via the Irg1/itaconate axis^37^. On the other hand, alanine increased, corroborating an increased glycolytic activity and reduced shunting of pyruvate into mitochondrial metabolism. Suppressed oxidative metabolism is also in line with the observed decrease in the ATP/ADP ratio, which favors enhanced glycolysis^38^ and has been previously observed in murine macrophages^34^. Moreover, the levels of nicotinamide adenine dinucleotide (NAD^+^), a cofactor involved in multiple biological processes including in redox reactions of glycolysis and mitochondrial respiration^39^, decreased in M1 macrophages, consistently with other studies^15,40^. Glutathione (GSH), a major intracellular antioxidant, also decreased upon pro-inflammatory stimulation, possibly to counteract reactive oxygen and nitrogen species produced by pro-inflammatory macrophages^41^. Taurine could also play an antioxidant role^42^ and/or act is osmotic regulation, together with *myo*-inositol, which can additionally be involved in phosphoinositide turnover and cell signaling. Finally, M1 macrophages presented increased levels of choline, glycerophosphocholine and phosphoethanolamine, which suggest breakdown of membrane phospholipids, as reported by others^25^.

### Flavonoids differentially modulate the metabolome of M1 pre-polarized macrophages

**Figure 3:**
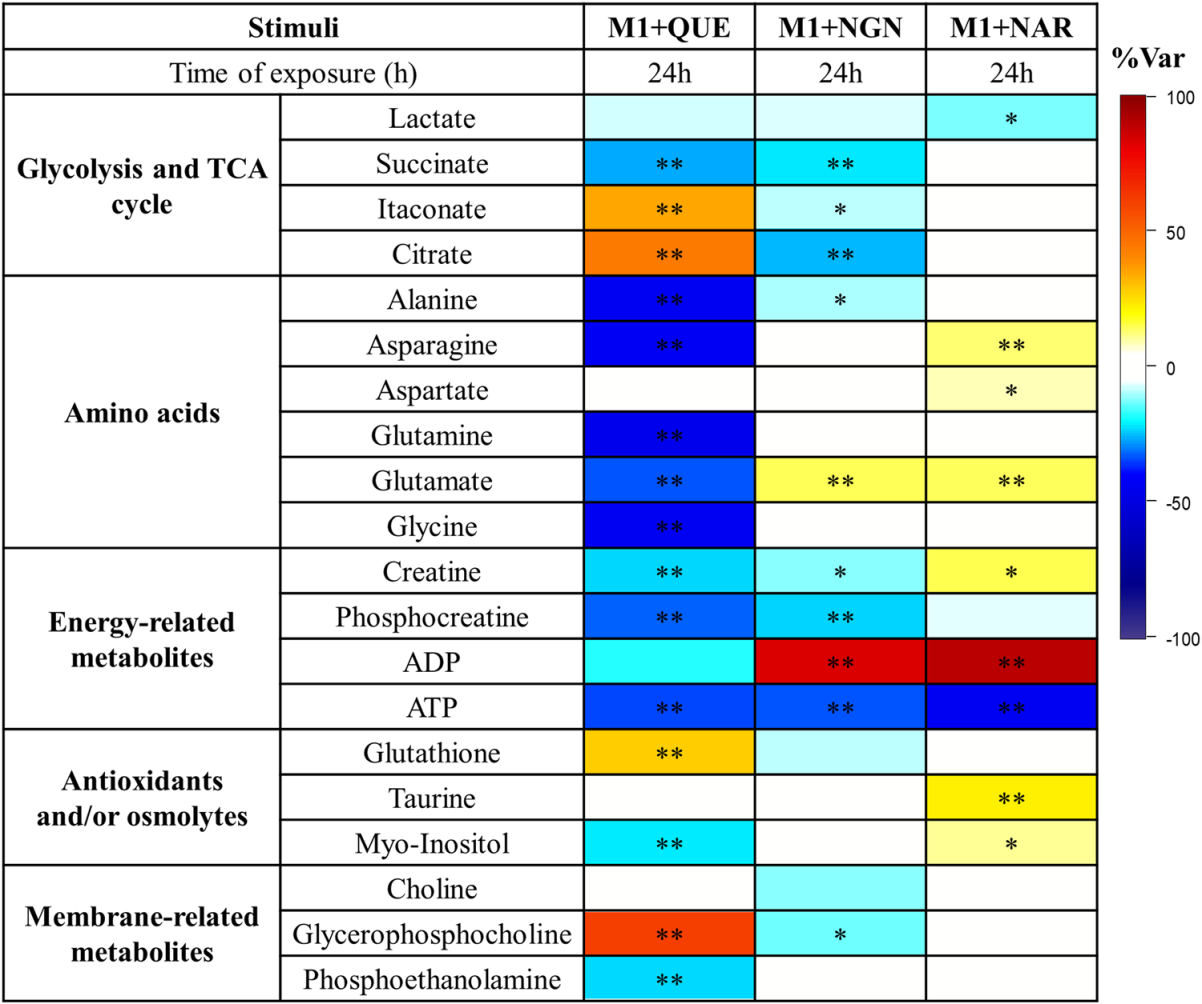
Heatmap of metabolic effects induced by each flavonoid, where the color scale represents the percentage of variation in M1 + flavonoid *vs*. M1-Ct macrophages (*p < 0.05, **p < 0.01). This Figure was generated using R software version 3.4.1, R Core Team (2017). R: A language and environment for statistical computing. R Foundation for Statistical Computing, Vienna, Austria. http://www.R-project.org/.

The three flavonoids tested were found to alter the endometabolome of M1 pro-inflammatory macrophages in distinct ways. The PCA scores scatter plot obtained for control and flavonoid-incubated samples (Fig. 2B) suggests that the flavonoid with the highest metabolic impact (scores far away from controls) was quercetin, whereas naringin-incubated samples were less separated from controls. This was confirmed through PCA and PLS-DA pairwise comparisons (Fig. 2C), as higher/lower Q^2^ values and more/less intense loadings coloring were obtained for the discrimination between controls and quercetin/naringin-treated cells. Assessment of individual metabolite variations (Fig. 3) revealed the features of each metabolic signature (detailed in Supporting Table S2). Metabolic effects common to all flavonoids were confined to decreased lactate and ATP levels. On the other hand, both quercetin and naringenin promoted a decrease in succinate, alanine, creatine and phosphocreatine, whereas naringenin and naringin-treated macrophages displayed increased glutamate and ADP levels in relation to controls (M1-Ct). An increased intracellular ADP:ATP ratio is recognized as an indicator of cell death^38^. However, in the present work, in spite of the relative decrease in ATP and the concomitant increase in ADP (observed in naringenin- and naringin-treated cells), the viability of macrophages incubated for 24 h with flavonoids was not compromised. These results suggest that intracellular ATP levels did not reach values that were critical to cell survival. On the other hand, it is well known that an increase in ADP:ATP may activate the adenosine monophosphate-activated protein kinase (AMPK) - a major regulator of glucose and lipid metabolism^43^, consequently inhibiting anabolic reactions and stimulating catabolic pathways. Indeed, previous works have highlighted the ability of several phytochemicals to activate AMPK. In particular, quercetin has been reported to modulate activation of murine bone marrow-derived macrophages (BMDM) through the AMPK/SIRT1 pathway^44^, and Manuka honey, of which quercetin is a major polyphenolic component, was found to activate AMPK in LPS-stimulated murine macrophages, improving mitochondrial respiration^45^. Moreover, quercetin and other flavonoids are believed to confer mitochondrial protection in different cell types by activating signalling pathways related to antioxidant defences^46^. Hence, correlations between the metabolome modulation hereby described and signalling pathways related to energy sensing and oxidative stress responses should be investigated in future studies.

### Flavonoids attenuate pro-inflammatory cytokines and reverse some M1-related metabolic changes

**Figure 4:**
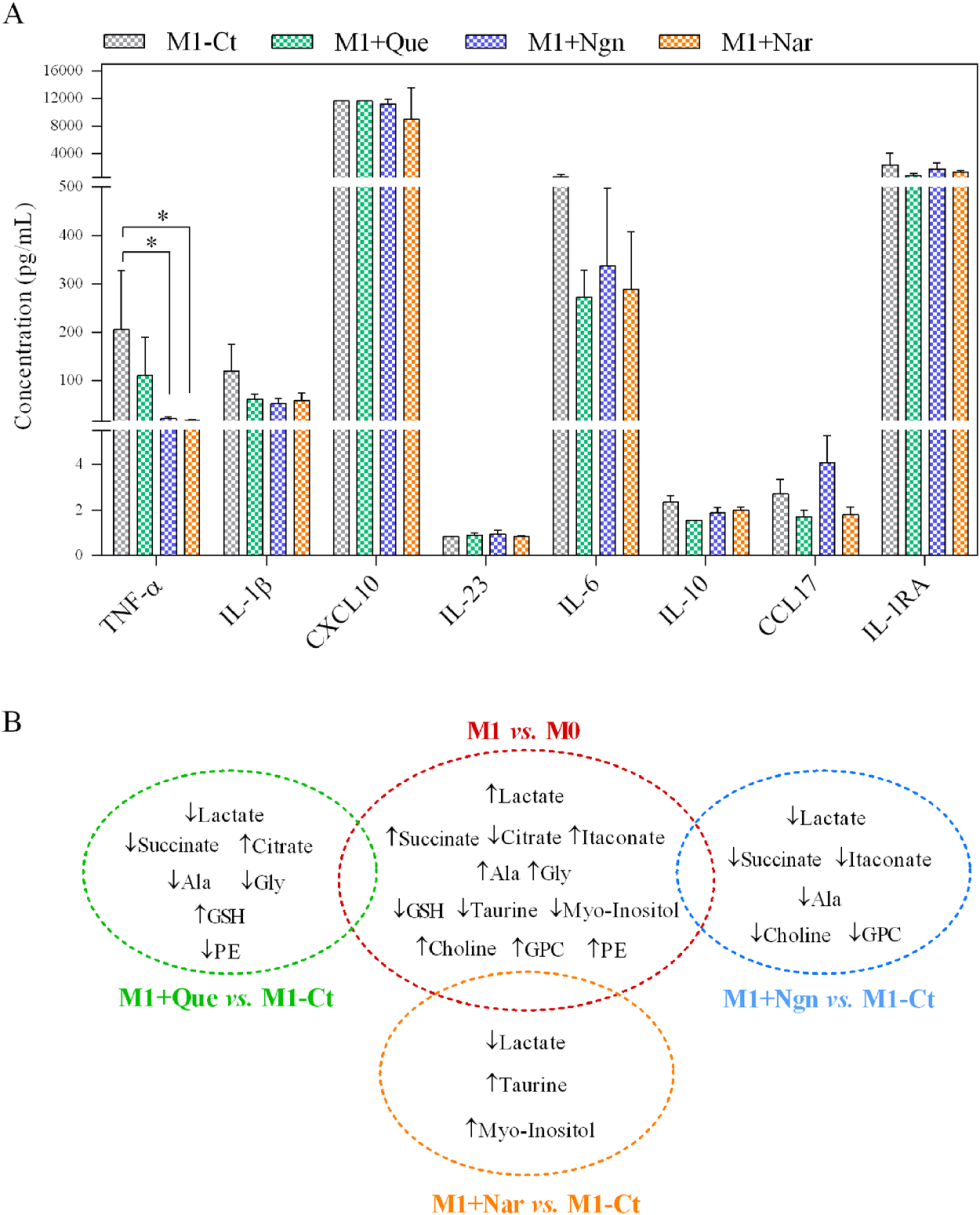
(**A**) Levels of cytokines quantified in the supernatants of pre-polarized M1 macrophages incubated for additional 24 h with fresh medium (M1-Ct) or with fresh medium containing quercetin (Que), naringenin (Ngn) or naringin (Nar); IL-12p40 and IL-12p70 below the limit of detection (not shown); The graph was generated using GraphPad Prism trial version 7.00, GraphPad Software, La Jolla California USA, www.graphpad.com; (**B**) Summary of metabolic variations in M1 *vs*. M0 macrophages that were reverted by each of the studied flavonoids.

The three flavonoids studied, especially naringenin and naringin, considerably reduced the production of the pro-inflammatory factor TNF-α by pre-polarized M1 macrophages, and showed a trend for attenuating the levels of IL-1β and IL-6 (Fig. 4A). Additionally, naringenin caused a slight (non-significant) increase in the anti-inflammatory chemokine CCL17, also known as thymus- and activation-regulated chemokine (TARC). In line with these results, decreased expression of several inflammation-related genes has been reported for quercetin-treated THP-1-derived macrophages^47,48^, as well as for macrophages incubated with either naringenin^49,50^ or naringin^51,52^.

**Figure 5:**
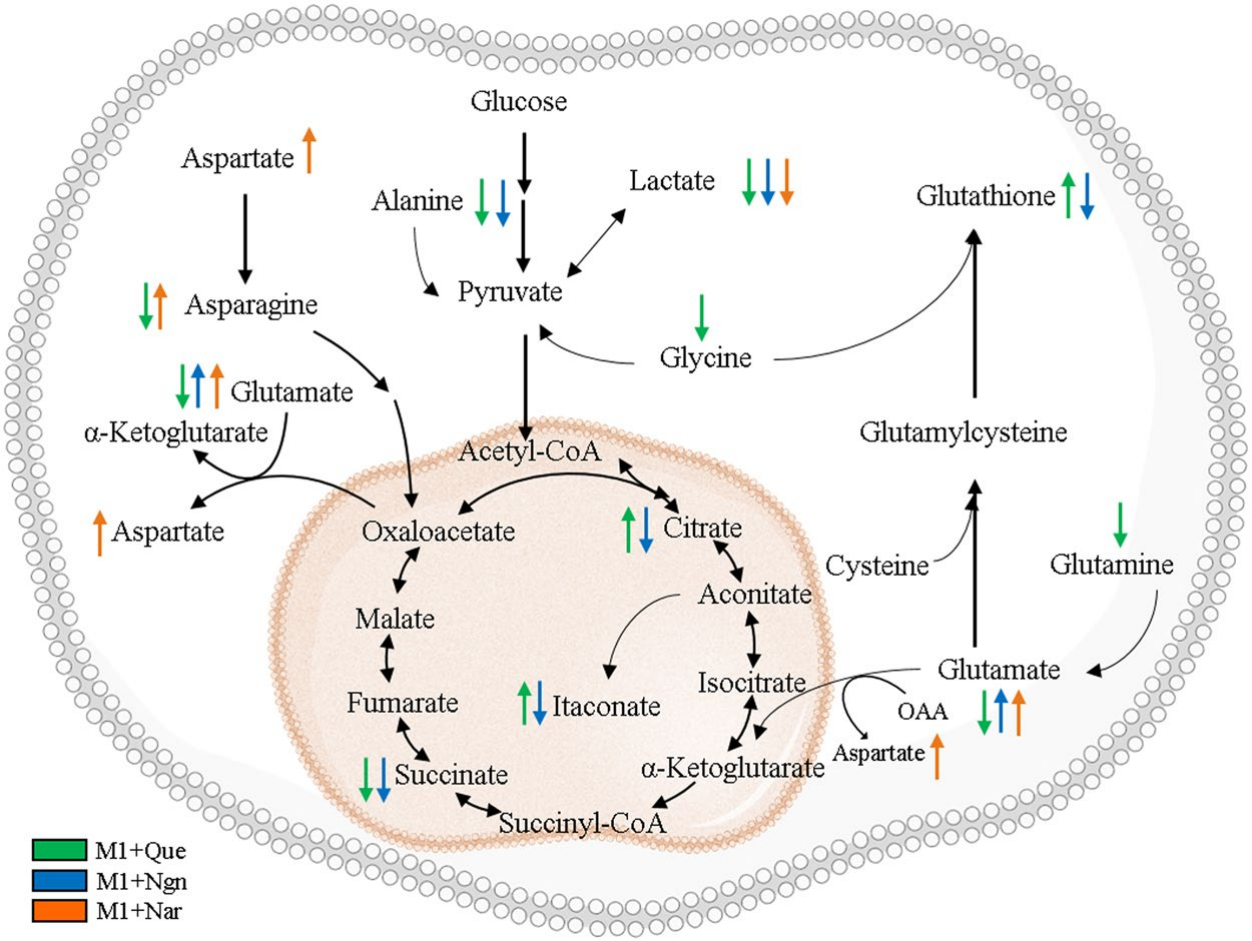
Overview of flavonoid-induced metabolic reprogramming of pro-inflammatory macrophages. Figure prepared using Microsoft PowerPoint 2013. Components of this figure were created using SMART Servier Medical Art templates, which are licensed under a Creative Commons Attribution 3.0 Unported License; https://smart.servier.com.

In order to cross-correlate the flavonoid metabolic signatures (described in the previous section) with their anti-inflammatory action, the ability of each flavonoid to revert the changes induced by M1 polarization was assessed. The Venn diagram displayed in Fig. 4B summarizes the results of this global comparison. Unlike M1 macrophages, flavonoid-treated cells displayed decreased intracellular lactate, which suggests downregulation of glycolytic activity. Analysis of glucose and lactate levels in cells-conditioned medium further revealed that, unlike pre-polarized M1 cells (M1-Ct), flavonoid-treated cells did not take up any glucose from the medium during the 24 h incubation, as extracellular glucose levels remained similar to those in the acellular medium incubated under the same conditions (Supporting Information Fig. S5). On the other hand, lactate excretion was maintained or even slightly increased (in the case of naringenin-exposed macrophages). Therefore, the reduction of intracellular lactate in flavonoid-treated cells likely reflects the blockage of glucose uptake from the culture medium and the consequent hindering of its glycolytic conversion into lactate. This is in accordance with an early study showing that several flavonoids, including quercetin and naringenin blocked glucose uptake by myelocytic U937 cells^53^. In that study, however, the glycone naringin did not inhibit glucose uptake, in contrast with our results, which might relate to the different cell lines used as model and/or their activation state. This emphasizes the relevance of performing untargeted metabolomic profiling in different cell lines to evaluate cell-specific flavonoid effects. Furthermore, it should be noted that the inhibitory activity of quercetin towards glucose transporters and glycolytic enzymes has also been widely reported in cancer cells^54^, which typically present a high glycolytic profile, similarly to pro-inflammatory macrophages. TCA cycle-related metabolites were differentially modulated by the three flavonoids. Quercetin-treated macrophages displayed decreased succinate and increased citrate levels, counteracting the effects of M1 polarization. On the other hand, the levels of the anti-inflammatory metabolite itaconate were sustained when pre-polarized M1 macrophages were incubated with quercetin (producing an increase in comparison to M1-Ct cells). These findings strongly suggest that quercetin was able to revert the TCA cycle pro-inflammatory reprogramming. Such effect was only mildly observed for naringenin and totally absent in the case of naringin. Regarding amino acids, opposite variations between M1 polarization and flavonoid incubation of M1 pre-polarized macrophages were found for alanine (in the case of quercetin and naringenin) and for glycine (in the case of quercetin). The strong impact of quercetin on amino acids could reflect their intensified use to replenish the TCA cycle and, in this way, a compensatory adaptation to cope with the lower glucose uptake. Another noticeable effect regarded GSH variation. While this metabolite decreased upon M1 polarization, pre-polarized M1 macrophages incubated with quercetin displayed increased GSH levels compared to M1-Ct, indicating enhanced antioxidant protection. Accordingly, the antioxidant action of quercetin in the human monoblastic cell line U937 was found to be favored in the presence of a large GSH pool^55^. Furthermore, naringin reverted the M1-related variations in taurine and myo-inositol, which have antioxidant and/or osmoregulatory roles, whereas naringenin differentially affected the levels of choline and glycerophosphocholine. Since choline is an essential component of major membrane phospholipids and glycerophosphocholine is a degradation product of phosphatidylcholine, their altered levels may possibly reflect membrane modification. Overall, it is clear that flavonoids had a wide range of metabolic effects on pro-inflammatory macrophages, which reflect their ability to influence different metabolic pathways, as summarized in Fig. 5.

## Conclusions

In this work, modulation of the metabolome of pro-inflammatory human macrophages by three different flavonoids has been reported for the first time. The results revealed several flavonoid-induced metabolic variations that were opposite to the pro-inflammatory metabolic reprogramming elicited by LPS + IFN-γ stimulation, thus suggesting their greater involvement in flavonoid-mediated immunomodulatory activity. In particular, quercetin, naringenin and naringin appeared to elicit differential interference with glycolysis, TCA cycle, antioxidant defenses and membrane composition, with quercetin inducing the most pronounced effects. Future molecular studies on specific metabolic enzymes and signaling pathways are warranted to help explaining the metabolomics findings.

## Supplementary information

Supplementary information

## Data Availability

The datasets generated during and/or analyzed during the current study are available from the corresponding authors on reasonable request.
