# Supplementary material for: Flavonoid-mediated immunomodulation of human macrophages involves key metabolites and metabolic pathways
Source: Sci Rep. 2019 Oct 17;9:14906. doi: 10.1038/s41598-019-51113-z (PMC6797761; doi:10.1038/s41598-019-51113-z)
Supplement: Supplementary file 1 — Supplementary information [file 41598_2019_51113_MOESM1_ESM.pdf]

Supporting Information

Flavonoid-mediated immunomodulation of human macrophages involves key metabolites and metabolic pathways

Luís F. Mendes, Vítor M. Gaspar, Tiago A. Conde, João F. Mano*, Iola F. Duarte*

CICECO – Aveiro Institute of Materials, Department of Chemistry, University of Aveiro, 3810-193 Aveiro, Portugal.

*Corresponding authors: João F. Mano, jmano@ua.pt; Iola F. Duarte, ioladuarte@ua.pt

Figure S1. Chemical structures of the flavonoids studied in this work: quercetin (a flavonol), naringenin (a flavanone) and naringin (a naringenin 7-O-glycoside).

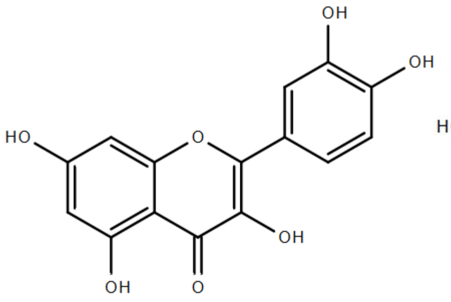

Quercetin

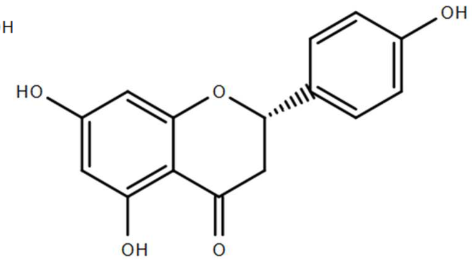

Naringenin

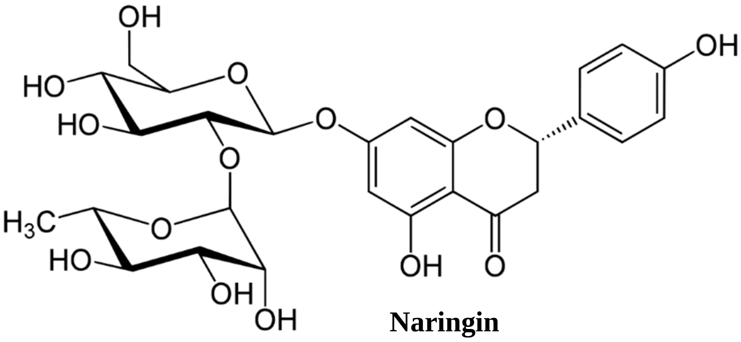

Naringin

Figure S2. Cytokines concentration in the culture medium of polarized M1 macrophages (red) and their respective controls (grey). Statistical significance was assessed using a one-way ANOVA, with a Sidak multiple comparisons test (n=3). * p-value < 0.05 ** p-value < 0.01.

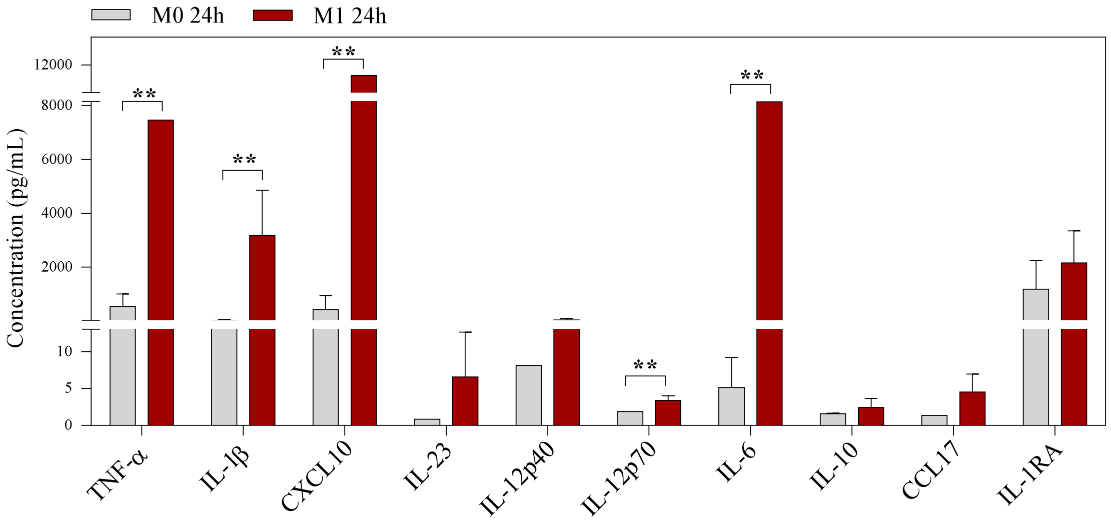

Figure S3. Cell viability of THP-1 derived macrophages exposed to quercetin, naringenin and naringin, for 24h, at concentrations ranging from 20 µM to 200 µM, as assessed by the Alamar Blue® reduction assay. Statistical significance assessed in each concentration of the compound against the control (grey bar) using a one-way ANOVA, with a Sidak multiple comparisons test (*n*=5). * p-value < 0.05 ** p-value < 0.01. K- represents control untreated cells.

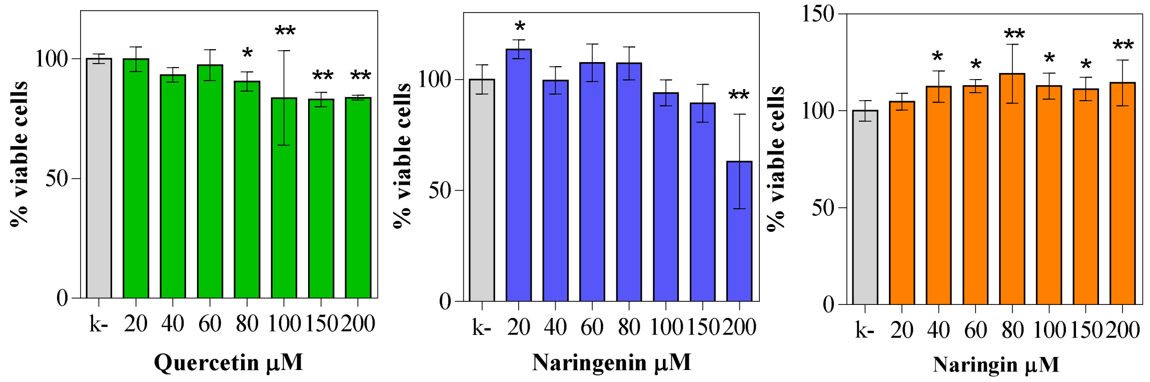

Figure S4. Expansions of (A) ^{1}H-^{1}H TOCSY and (B) *J*-resolved spectra of a polar extract from M0 macrophages. Signals are numbered in accordance with Table S1.

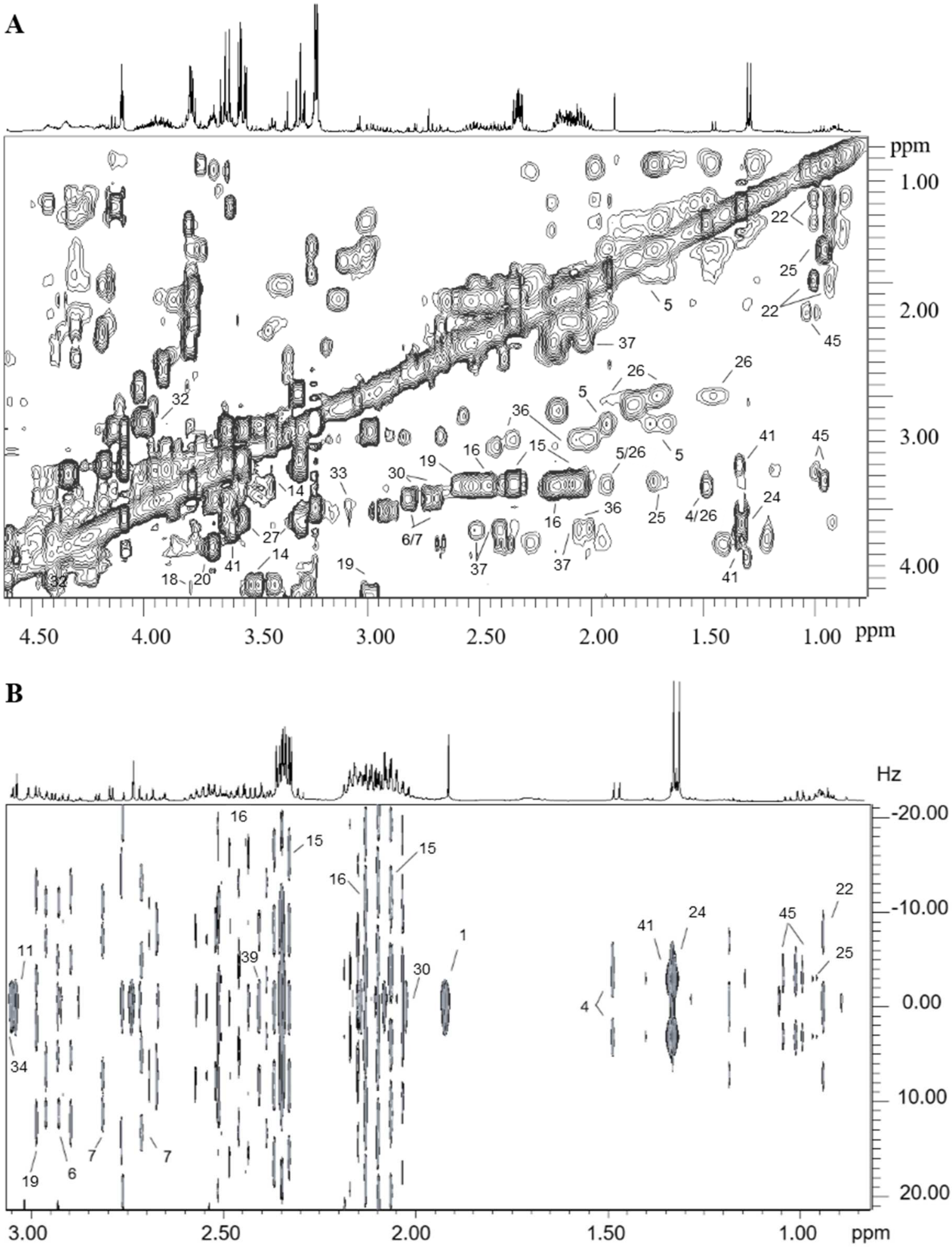

Figure S5. Extracellular and intracellular relative levels of glucose and lactate in pre-polarized M1 macrophages incubated for additional 24h with fresh medium (M1-Ct) or with fresh medium containing quercetin (Que), naringenin (Ngn) or naringin (Nar). The data were obtained through spectral integration of ^{1}H NMR signals. Statistical significance (* $p < 0.05$, ** $p < 0.01$) was assessed in relation to M1-Ct samples. Acellular medium represents complete culture medium without cells.

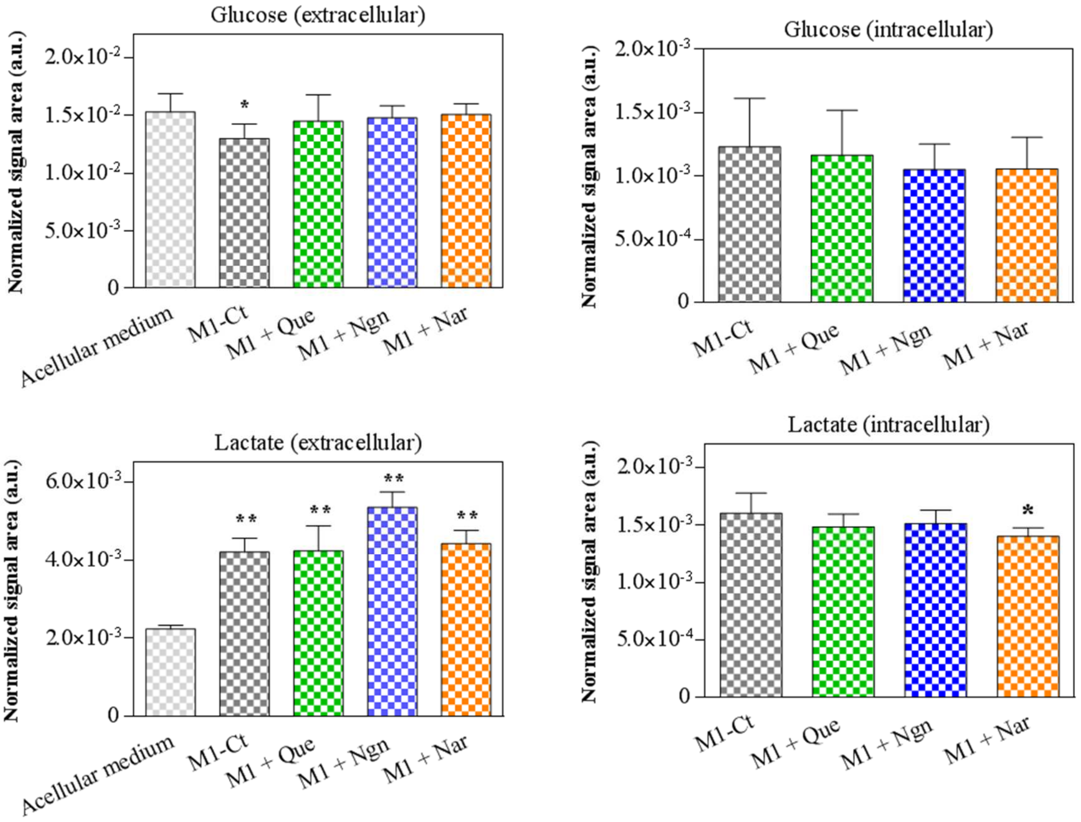

Table S1. Assignment of resonances in the ^{1}H-NMR profile of polar extracts from THP-1 derived macrophages. Multiplicity: s, singlet; d, doublet; t, triplet; q, quartet; m, multiplet; dd, doublet of doublets; dt, doublet of triplets; td, triplet of doublets.

No.	Compound	δ ^{1}H in ppm (multiplicity, assignment)
**1**	Acetate	1.926 (s, β-CH_3)
**2**	Acetone	2.236 (s, CH_3)
**3**	ADP	4.235 (m, C5'H, ribose); 4.40 (m, C4'H, ribose); 4.60 (m, C2'H, ribose); 6.155 (d, C1'H, ribose); 8.285 (s, C8H, ring); 8.53 (s, C2H, ring)
**4**	Alanine	1.487 (d, β-CH_2); 3.781 (q, α-CH)
**5**	Arginine	1.659 (m, γ-CH_2); 1.92 (m, β-CH_2); 3.254 (t, δ-CH_2); 3.784 (t, α-CH)
**6**	Asparagine	2.881 (m, β-CH); 2.931 (m, β'-CH); 4.012 (dd, α-CH)
**7**	Aspartate	2.672 (dd, β-CH); 2.802 (dd, β'-CH); 3.903 (dd, α-CH)
**8**	ATP	4.222 (m, C5'H, ribose); 4.296 (m. C5''H, ribose); 4.41 (m, C4'H, ribose); 4.61 (m, C2'H, ribose); 6.155 (d, C1'H, ribose); 8.825 (s, C8H, ring); 8.534 (s, C2H, ring)
**9**	Choline	3.214 (s, $N(CH_3)_3$); 3.523 (m, CH_2 (NH)); 4.067 (m, CH_2(OH))
**10**	Citrate	2.536 (d, α-CH_2 / β-CH_2); 2.662 (d, α'-CH_2 / β'-CH_2)
**11**	Creatine	3.039 (s, CH_3); 3.939 (s, N-CH_3)
**12**	Formate	8.465 (s, CH)
**13**	Fumarate	6.526 (s, CH)

No.	Compound	δ ^{1}H in ppm (multiplicity, assignment)
**14**	Glucose	3.239 (dd, C3H); 3.405 (m, C5H); 3.464 (m, C6H); 3.535 (dd, C3H); 3.72 (m, C4H, C11H); 3.827 (m, C6H, C11H); 3.895 (dd, C11H); 4.658 (d, C2H); 5.235 (d, C2H)
**15**	Glutamate	2.044 (m, β-CH); 2.12 (β'-CH); 2.333 (m, γ-CH_2); 3.77 (dd, α-CH)
**16**	Glutamine	2.142 (m, β-CH_2); 2.454 (m, γ-CH_2); 3.78 (t, α-CH)
**17**	Glycine	3.575 (s, α-CH_2)
**18**	Glutathione, oxidized (GSSG)	2.17 (m, β-CH_2, Glu); 2.535 (m, γ-CH_2, Glu); 2.988 (m, β-CH_2, Cys); 3.314 (m, β-CH_2, Cys'); 3.783 (m, α-CH_2, Gly); 3.783 (m, α-CH, Glu)
**19**	Glutathione, reduced (GSH)	2.175 (m, β-CH_2, Glu); 2.564 (m, γ-CH_2, Glu); 2.96 (m, β-CH_2, Cys); 3.783 (m, α-CH, Gly); 4.603 (q, α-CH, Cys)
**20**	Glycerophosphocholine	3.24 (s, $N(CH_3)_3$); 3.675 (m, β'-CH_2(N) / γ-CH_2(OH)); 3.917 (s, α-CH_2 / β-CH_2); 4.326 (m, α'-CH_2(P))
**21**	Histidine	3.232 (m, β-CH_2); 4.00 (m, α-CH_2); 7.165 (s, C4H, ring); 8.072 (s, C2H, ring)
**22**	Isoleucine	0.944 (t, δ-CH_3); 1.013 (d, β-CH_3); 1.258 (m, γ-CH_2); 1.470 (m, γ'-CH_2); 1.984 (m, β-CH); 3.673 (d, α-CH)
**23**	Itaconate	3.158 (m, α-CH_2), 5.382 (m, CH_2); 5.860 (m, CH_2')
**24**	Lactate	1.329 (d, β-CH_3); 4.125 (q, α-CH)
**25**	Leucine	0.963 (t, δ-CH_3); 1.699 (m, γ-CH / β-CH_2); 3.736 (m, α-CH)
**26**	Lysine	1.439 (m, γ-CH_2); 1.725 (m, δ-CH_2); 1.904 (m, β-CH_2); 3.001 (t, ε-CH_2); 3.765 (t, α-CH)

No.	Compound	δ ^{1}H in ppm (multiplicity, assignment)
**27**	Methylguanidine	2.845 (s, CH_3(N))
**28**	*myo*-Inositol	3.286 (t, C5H); 3.544 (dd, C1'H / C3H); 3.632 (t, C4H / C6H); 4.072 (t, C2H)
**29**	NAD+	4.230 (m, A5'H); 4.357 (m, A4'H); 4.383 (m, A4'H / N5'H); 4.411 (dd, N3'H); 4.469 (m, A3'H); 4.513 (m, N2'H); 6.031 (d, N1'H); 6.104 (d, A1'H); 8.172 (s, A2H / N5H); 8.827 (d, N4H); 9.122 (d, N6H); 9.339 (s, N2H)
**30**	*N*-Acetylaspartate	2.030 (s, CH_3); 2.507 (dd, β-CH_2); 2.69 (dd, β'-CH_2); 4.398 (dd, α-CH)
**31**	Pantothenate	0.905 (s, CH_3); 0.925 (s,CH_3); 2.435 (t, α-CH_2); 3.416 (d, CH_2); 3.438 (q, β-CH_2); 3.510 (d, CH_2); 3.99 (s, CH)
**32**	Phenylalanine	3.146 (m, β-CH); 3.280 (dd, β'-CH); 4.00 (m, α-CH); 7.384 (d, C2H / C6H, ring); 7.39 (d, C4H, ring); 7.43 (t, C3H / C5H, ring)
**33**	Phosphocholine	3.23 (s, N$(CH_3)_3$); 3.62 (m, N-CH_2); 4.172 (m, PO_3-CH_2)
**34**	Phosphocreatine	3.04 (s, CH_3); 3.955 (s, CH_2)
**35**	Phosphoethanolamine	3.232 (t, CH_2(O)); 4.005 (td, N-CH_2)
**36**	Proline	2.014 (m, γ-CH_2); 2.08 (m, β-CH); 2.35 (m, β'-CH); 3.35 (dt, δ-CH); 3.418 (dt-δ'-CH); 4.137 (dd, α-CH)
**37**	Pyroglutamate	2.05 (m, β-CH_2); 2.40 (m, γ-CH_2); 2.495 (m, β'-CH_2); 4.17 (dd, α-CH)
**38**	Serine	3.852 (dd, α-CH); 3.99 (m, β-CH_2)
**39**	Succinate	2.41 (s, CH_2)

No.	Compound	δ ^{1}H in ppm (multiplicity, assignment)
**40**	Taurine	3.27 (t, S-CH_2); 3.43 (t, N-CH_2)
**41**	Threonine	1.341 (d, γ-CH_3); 3.950 (d, α-CH); 4.269 (m, β-CH)
**42**	Tyrosine	3.07 (m, β'-CH); 3.21 (m, β-CH); 3.95 (m, α-CH); 6.91 (d, C3H / C5H, ring); 7.21 (d, C2H / C6H, ring)
**43**	UDP	4.231 (m, C5'H, ribose); 4.280 (m, C4'H, ribose); 4.398 (t, C2'H, ribose); 4.44 (t, C3'H, ribose); 5.97 (s, C1'H, ribose); 5.98 (d, C6H, ring); 7.995 (d, C5H, ring)
**44**	UTP	4.26 (m, C5'H, ribose); 4.295 (m, C4'H, ribose); 4.416 (t, C2'H, ribose); 4.45 (t, C3'H, ribose); 5.98 (s, C1'H, ribose); 5.995 (d, C6H, ring); 7.982 (d, C5H, ring)
**45**	Valine	1.027 (d, γ-CH_2); 2.265 (m, β-CH); 3.625 (d, α-CH)

Table S2: Main metabolite variations in the polar extracts of pro-inflammatory M1 macrophages and in flavonoid-treated pre-polarized M1 macrophages. The variations are expressed as % of variation (%Var) with respective error (±), effect size (ES) and *p*-value (*p*). Variations with |ES| < 0.5 were considered to be null.

		**M1 *vs.* M0**	**M1+Que *vs.* M1-Ct**	**M1+Ngn *vs.* M1-Ct**	**M1+Nar *vs.* M1-Ct**
**Glucose**	**%Var**	-16.95	0	0	0
	**±**	7.11			
	**ES**	-1.40	0	0	0
	p	0.02959			
**Lactate**	**%Var**	25.00	-7.51	-6.93	-12.59
	**±**	3.91	5.70	5.47	5.17
	**ES**	3.27	-0.73	-0.70	-1.39
	p	0.00059	0.20414	0.22408	0.03652
**Succinate**	**%Var**	95.14	-26.38	-21.51	0
	**±**	12.56	4.92	4.43	
	**ES**	3.22	-3.29	-2.90	0
	p	0.00506	0.00020	0.00096	
**Itaconate**	**%Var**	330.85	35.00	-8.78	0
	**±**	8.77	2.70	3.35	
	**ES**	9.12	5.88	-1.46	0
	p	0.00006	0.000002	0.02254	
**Citrate**	**%Var**	-18.43	44.81	-25.32	0
	**±**	4.55	6.16	5.01	
	**ES**	-2.56	3.17	-3.08	0
	p	0.00389	0.00024	0.00125	
**Alanine**	**%Var**	12.22	-44.01	-9.81	0
	**±**	4.58	4.64	4.07	
	**ES**	1.40	-6.49	-1.35	0
	p	0.03318	0.00001	0.03425	
**Asparagine**	**%Var**	0	-43.16	6.32	13.26
	**±**		4.33	2.73	2.43
	**ES**	0	-6.77	1.19	2.73
	p		0.0000002	0.05575	0.00201
**Aspartate**	**%Var**	0	0	0	9.04
	**±**				3.30
	**ES**	0	0	0	1.40
	p				0.02962
**Glutamine**	**%Var**	-14.29	-45.97	0	0
	**±**	4.85	4.47		
	**ES**	-1.79	-7.11	0	0
	p	0.01485	0.0000004		

		M1 *vs.* M0	M1+Que *vs.* M1-Ct	M1+Ngn *vs.* M1-Ct	M1+Nar *vs.* M1-Ct
**Glutamate**	**%Var**	0	-33.32	14.78	14.49
	**±**		4.19	3.83	2.98
	**ES**	0	-5.09	1.91	2.42
	p		0.000002	0.00568	0.00114
**Glycine**	**%Var**	14.77	-43.56	0	0
	**±**	7.52	4.92		
	**ES**	1.11	-6.03	0	0
	p	0.12892	0.00005		
**Leucine**	**%Var**	-17.89	0	0	0
	**±**	3.43			
	**ES**	-3.06	0	0	0
	p	0.00031			
**Isoleucine**	**%Var**	-21.86	0	0	0
	**±**	2.64			
	**ES**	-4.76	0	0	0
	p	0.00003			
**Valine**	**%Var**	-11.70	0	0	0
	**±**	3.52			
	**ES**	-1.91	0	0	0
	p	0.00659			
**Creatine**	**%Var**	0	-22.89	-11.67	15.22
	**±**		6.06	5.04	4.68
	**ES**	0	-2.27	-1.31	1.61
	p		0.00231	0.04816	0.01802
**Phosphocreatine**	**%Var**	0	-32.13	-23.09	0
	**±**		7.03	6.77	
	**ES**	0	-2.90	-2.05	0
	p		0.00214	0.00900	
**ADP**	**%Var**	65.90	-17.83	83.79	90.24
	**±**	25.99	11.94	13.04	11.17
	**ES**	1.15	-0.87	2.41	2.97
	p	0.10287	0.14166	0.00179	0.00033
**ATP**	**%Var**	-38.77	-34.51	-33.26	-43.75
	**±**	10.37	10.40	10.10	10.28
	**ES**	-2.65	-2.14	-2.10	-2.90
	p	0.00396	0.00524	0.00650	0.00216
**UTP**	**%Var**	-31.28	0	0	0
	**±**	6.31			
	**ES**	-3.02	0	0	0
	p	0.00046			
**NAD+**	**%Var**	-57.94	0	0	0
	**±**	8.41			
	**ES**	-4.77	0	0	0
	p	0.00008			

		**M1 *vs.* M0**	**M1+Que *vs.* M1-Ct**	**M1+Ngn *vs.* M1-Ct**	**M1+Nar *vs.* M1-Ct**
**Glutathione**	**%Var**	-21.77	28.40	-8.64	0
	**±**	10.16	3.77	3.94	
	**ES**	-1.37	3.51	-1.22	0
	p	0.05327	0.00008	0.05549	
**Taurine**	**%Var**	-16.46	0	0	22.50
	**±**	8.85			2.41
	**ES**	-1.19	0	0	4.47
	p	0.09731			0.00018
**Myo-Inositol**	**%Var**	-17.04	-21.33	0	11.10
	**±**	7.35	5.05		4.07
	**ES**	-1.44	-2.52	0	1.38
	p	0.03930	0.00228		0.04294
**Choline**	**%Var**	33.58	0	-11.74	0
	**±**	9.75		5.76	
	**ES**	1.69	0	-1.21	0
	p	0.01776		0.05847	
**Glycerophosphocholine**	**%Var**	23.74	61.58	-13.41	0
	**±**	6.51	4.88	5.32	
	**ES**	1.99	5.14	-1.44	0
	p	0.02537	0.000002	0.02712	
**Phosphoethanolamine**	**%Var**	17.30	-22.63	0	0
	**±**	1.68	2.82		
	**ES**	5.27	-4.82	0	0
	p	0.00001	0.00001		
